# Identifying microbe-disease association based on graph convolutional attention network: Case study of liver cirrhosis and epilepsy

**DOI:** 10.3389/fnins.2022.1124315

**Published:** 2023-01-19

**Authors:** Kai Shi, Lin Li, Zhengfeng Wang, Huazhou Chen, Zilin Chen, Shuanfeng Fang

**Affiliations:** ^1^College of Information Science and Engineering, Guilin University of Technology, Guilin, China; ^2^Guangxi Key Laboratory of Embedded Technology and Intelligent System, Guilin University of Technology, Guilin, China; ^3^College of Science, Guilin University of Technology, Guilin, China; ^4^Department of Developmental and Behavioural Pediatric Department & Department of Child Primary Care, Xinhua Hospital, Shanghai Jiao Tong University School of Medicine, Shanghai, China; ^5^Department of Children Health Care, Children’s Hospital Affiliated to Zhengzhou University, Zhengzhou, China

**Keywords:** gut-liver-brain axis, microbe-disease associations, similarity network, graph convolutional network, graph attention network, liver cirrhosis, epilepsy

## Abstract

The interactions between the microbiota and the human host can affect the physiological functions of organs (such as the brain, liver, gut, etc.). Accumulating investigations indicate that the imbalance of microbial community is closely related to the occurrence and development of diseases. Thus, the identification of potential links between microbes and diseases can provide insight into the pathogenesis of diseases. In this study, we propose a deep learning framework (MDAGCAN) based on graph convolutional attention network to identify potential microbe-disease associations. In MDAGCAN, we first construct a heterogeneous network consisting of the known microbe-disease associations and multi-similarity fusion networks of microbes and diseases. Then, the node embeddings considering the neighbor information of the heterogeneous network are learned by applying graph convolutional layers and graph attention layers. Finally, a bilinear decoder using node embedding representations reconstructs the unknown microbe-disease association. Experiments show that our method achieves reliable performance with average AUCs of 0.9778 and 0.9454 ± 0.0038 in the frameworks of Leave-one-out cross validation (LOOCV) and 5-fold cross validation (5-fold CV), respectively. Furthermore, we apply MDAGCAN to predict latent microbes for two high-risk human diseases, i.e., liver cirrhosis and epilepsy, and results illustrate that 16 and 17 out of the top 20 predicted microbes are verified by published literatures, respectively. In conclusion, our method displays effective and reliable prediction performance and can be expected to predict unknown microbe-disease associations facilitating disease diagnosis and prevention.

## 1. Introduction

Microbes are mainly categorized as bacteria, fungi, archaea and viruses, which inhabit all parts of the human body, but the greatest number of microbes are found in the gut (Blum, 2017; Kitamoto et al., 2020; de Vos et al., 2022). Gut microbiota plays an important role in regulating host physiological processes (e.g., immunity and metabolism), and its ecological disorders are closely related to the brain, liver and other organs (Gonzalez-Ochoa et al., 2017; Tooley, 2020; Gabanyi et al., 2022). Recently, increasing medical studies reported that the gut-liver-brain axis plays a fundamental role in the pathogenesis of various diseases (Won et al., 2022), which is the bidirectional relationship between the gut and its microbiota, the liver, and the brain. Besides, gut microbiota exert their actions at different levels of the gut-liver-brain axis, impacting disease progression via changing gut-liver-brain axis communication (Fuenzalida et al., 2021). For example, liver cirrhosis is a common chronic progressive liver disease with high mortality caused by one or more factors, such as alcohol, metabolic disorders, drugs and so on (Gan et al., 2022). Researchers found out that the gut microbiota is a key factor in the progression of chronic liver disease, while the gut microbiota (e.g., *Enterococcus* and *Escherichia coli*) in patients with liver cirrhosis has significant changes compared to healthy individuals (Hussain et al., 2020; Ren et al., 2021). Moreover, *Escherichia coli* can produce an active amino acid GABA through the metabolic pathway (Altaib et al., 2022), which can activate glucose metabolism in the brain, improve brain function and impact epileptic seizures via the genetic pathway (Feng et al., 2022). Epilepsy is another of the third most common chronic neurological disorder worldwide, which usually suffers from depression, anxiety, obsessive-compulsive disorder, migraine and other disorders (Löscher et al., 2020). Many underlying disease mechanisms can lead to epilepsy, and the cause of the disease remains unknown. Research results have revealed that intestinal microbial imbalance can impact the occurrence of epilepsy due to the close relationship between the central nervous system and the gastrointestinal tract (Al-Beltagi and Saeed, 2022). For instance, serotonin produced by Enterococcus is a neurotransmitter in the central and peripheral nervous systems and has a certain inhibitory effect on the seizure of epilepsy (Deidda et al., 2021). Hence, studying disease-associated microbes not only advances the understanding of their pathogenesis, but also provides many new medical strategies for diseases. However, traditional biological experiments are difficult to meet the requirements of biomedical research owing to complex processes and expensive cost. Therefore, it is essential to develop efficient new prediction algorithms for microbe-disease association prediction.

Current computational methods for microbe-disease association prediction can be primarily classified as path-based methods, network-based methods and feature learning methods. Path-based methods usually calculate the microbe-disease association probability based on the number and weighted scores of various types of paths between two nodes. Chen et al. (2017) proposed the first computational method for microbe-disease association prediction based on the katz measure, which identified the microbe-disease correlation by calculating all paths of different lengths between microbes and diseases. Long and Luo (2019) calculated the probability score of microbe-disease pairs based on a weighted meta-graph search algorithm on a heterogeneous network to find possible microbe-disease associations. Network-based methods infer prospective microbe-disease associations through information propagation in a heterogeneous network. Yin et al. (2020) employed the structural similarity information of biological entities of diseases and microbes, combining spatial projection and label propagation to predict unknown microbe-disease associations. Yang et al. (2021) designed a novel identification method based on multi-similarities bilinear matrix factorization to find possible microbe-disease associations on a heterogeneous network. Yin et al. (2022) used the multiple kernel learning method to fuse similarities of microbe and disease, and then used the label propagation method to make predictions for disease-related potential microbes. Feature learning methods automatically extract features or representations from data through the model, and then reconstruct new microbe-disease associations by the features. Li et al. (2020) raised a neural network approach based on the backpropagation of a modified hyperbolic tangent activation function to predict disease-related microbes. Wang et al. (2021) applied random walk and graph embedding algorithm LINE to preserve graph structure through first-order and second-order proximity and to learn the latent feature representations of microbes and diseases, afterward obtained new microbe-disease associations by refactoring the representation. Long et al. (2021) developed an embedding representation method based on inductive matrix completion and graph attention network to infer the possible associations between microbes and diseases. Although the previous methods have achieved prominent results, more effective methods still need to be developed to screen latent microbe-disease associations.

In this study, we propose a deep learning framework to predict microbe-disease association, which combines the graph convolutional network and the graph attention network. First, we construct an informative heterogeneous network composed of the known microbe-disease association network and integrated multi-similarity networks, which fuse the Gaussian kernel similarity network and functional similarity network of microbe and disease, respectively. Then, MDAGCAN learns the feature representation of each node with the information of its neighbors and itself in the heterogeneous network by multi-layer graph convolution. Subsequently, the node representations serve as the input of graph attention layers. In graph attention layers, the node representations learned from graph convolutional layers further are enhanced by aggregating the weighted sum of neighbors’ information. Ultimately, the unknown microbe-disease associations are reconstructed by a bilinear decoder. In addition, our method compares with state-of-the-art methods on the datasets HMDAD and MASI and is applied to the prediction of associated microbes in liver cirrhosis and epilepsy. The results confirm that our model is effective and reliable for inferring potential microbe-disease associations.

## 2. Materials

### 2.1. Human microbe-disease associations

In this work, we download two public databases of known microbe-disease association HMDAD^1^ (Ma et al., 2017) and MASI^2^ (Zeng et al., 2021). HMDAD is the most frequently utilized human microbe-disease association database containing 450 non-redundant associations between 292 microbes and 39 diseases, and MASI covers microbial composition changes in different types of diseases with 629 associations involving 123 microbes and 56 diseases. The detailed statistics of the two microbe-disease association datasets above are exhibited in Table 1.

**TABLE 1:** The overall statistics for the microbe-disease association dataset.

Dataset	Microbe	Disease	Associations
HMDAD	292	39	450
MASI	123	56	629

The microbe-disease association is represented as a binary adjacent matrix **A** ∈ ℝ^*nd*×*nm*^, where **A**_**i***j*_ = 1 if there is an interaction between disease *d_i_* and microbe *m_i_*, otherwise **A**_**i***j*_ = 0.

## 3. Methods

**FIGURE 1:**
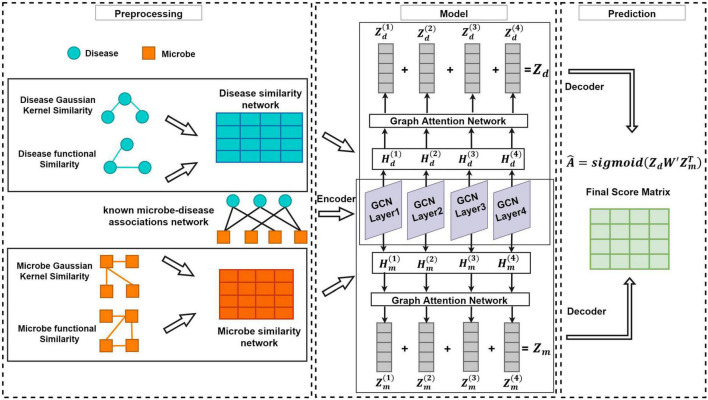
The flowchart of MDAGCAN for novel microbe-disease association prediction.

As shown in the flowchart of MDAGCAN (Figure 1), we introduce a graph convolutional attention network model to identify latent microbe-disease associations, which combines the graph convolutional network and graph attention network. MDAGCAN works in three stages to make predictions. Firstly, we construct a heterogeneous network consisting of a known microbe-disease association network, an integrated disease similarity network, and an integrated microbe similarity network. Secondly, latent representations of microbes and diseases are encoded and learned by graph convolutional layers and graph attention layers. Finally, MDAGCAN leverages a bilinear decoder to obtain the final association scores of microbe-disease pairs.

### 3.1. Similarity computation

#### 3.1.1. Gaussian interaction profile kernel similarity for microbe and disease

We calculate the Gaussian interaction profile kernel similarity of microbes according to the assumption that microbes with similar functions are more likely trend to connect similar diseases (Long et al., 2021). First, we present *GIP*(*m*_*i*_) as the interaction profile of the specific microbe *m_i_*, where it indicates the *i*th column of adjacent matrix **A**. Then, the Gaussian interaction profile kernel similarity *KM*(*m*_*i*_,*m*_*j*_) between microbe *m_i_* and *m_j_* can be defined as follows:


(1) $$K\,M\,\left(m_{i},m_{j}\right)=e\,x\,p\,\left(-\lambda_{m}\,{||G\,I\,P\,\left(m_{i}\right)-G\,I\,P\,\left(m_{j}\right)||}^{2}\right)$$

where λ_*m*_ indicates the normalized kernel bandwidth, the computation formula is below:


(2) $$\lambda_{m}=\frac{\lambda_{m}^{’}}{\frac{1}{n\,m}\,\sum_{t=1}^{n\,m}{||G\,I\,P\,\left(m_{t}\right)||}^{2}}$$

where $\lambda_{m}^{’}$ is the original bandwidth and is usually set to 1.

Similarly, we derive the Gaussian interaction profile kernel similarity between disease pairs, and construct the disease Gaussian interaction profile kernel similarity matrix *KD* ∈ ℝ^*nd*×*nd*^(0≤*KD*(*d*_*i*_,*d*_*j*_)≤1).

#### 3.1.2. Microbe functional similarity

Microbe functional similarity is calculated using a similar approach to Kamneva (2017), capturing the interactions between proteins encoded in the genomes of two microbes. The protein-protein functional interaction network is retrieved from the STRING v11 database^3^ to characterize the functional similarity of microbes by the similarity of microbial genomic proteins, and microbes with more common genes are more similar to each other. We use *FM*(*m*_*i*_,*m*_*j*_) to denote the functional similarity between microbe *m_i_* and microbe *m_j_*, where *FM* ∈ ℝ^*nm*×*nm*^.

#### 3.1.3. Disease functional similarity

In this work, we calculate disease functional similarity based on functional associations between disease-related genes with the assumption that similar diseases tend to interact with similar genes (Wei and Liu, 2020). We utilize the HumanNet v2.0 database (Hwang et al., 2019) to access gene interactions, where each interaction has a log-likelihood score (LLS) assessing the probability of a functional association between genes. For disease *d_i_* and disease *d_j_*, their functional similarity formula can be defined as follows:


(3) $$F\,D\,\left(d_{i},d_{j}\right)=\frac{\sum_{1\leq x\leq m}F\,S_{D}^{G^{b}}\,\left(g_{x}^{a}\right)+\sum_{1\leq y\leq n}F\,S_{D}^{G^{a}}\,\left(g_{y}^{b}\right)}{m+n}$$

where $F\,S_{D}^{G_{b}}\,\left(g_{x}^{a}\right)={m\,a\,x}_{1\leq y\leq n}\left(L\,L\,S\,\left(g_{x}^{a},g_{y}^{b}\right)\right)$ indicates the maximum functional correlation score between a gene $g_{x}^{a}$ and a gene set $G^{b}=\left\{g_{1}^{b},g_{2}^{b},,g_{n}^{b}\right\}$, and similarly $F\,S_{D}^{G_{a}}\,\left(g_{y}^{b}\right)={m\,a\,x}_{1\leq x\leq m}\left(L\,L\,S\,\left(g_{x}^{a},g_{y}^{b}\right)\right)$ expresses the maximum functional correlation score between a gene $g_{y}^{b}$ and a gene set $G^{a}=\left\{g_{1}^{a},g_{2}^{a},\ldots ,g_{m}^{a}\right\}$. $L\,L\,S\,\left(g_{x}^{a},g_{y}^{b}\right)$ is the normalization of the log-likelihood score. *G^a^* and *G^b^* are the gene sets associated with the disease *d_i_* and *d_j_*, separately.

### 3.2. Different similarities integration

It is not easy to achieve functional similarities between all diseases and microbes due to incomplete biology information (i.e., disease-related genes and microbial genomic proteins). To further improve similarities for diseases and microbes, we design a new strategy to integrate Gaussian kernel similarity and functional similarity. Specifically, if there is no functional similarity *FM* between microbe *m_i_* and *m_j_*, the integrated similarity between *m_i_* and *m_j_* is defined as *GM*, otherwise, it is equal to the linear combination of microbe Gaussian interaction profile kernel similarity *GM* and microbe functional similarity *FM*. Similarly, the integrated similarity of diseases can be calculated as follows:


(4) $$M\,S\,\left(m_{i},m_{j}\right)=\left\{ \begin{array}{c} G\,M\,\left(m_{i},m_{j}\right),i\,f\,F\,M\,\left(m_{i},m_{j}\right)=0\\ \mu \,G\,M\,\left(m_{i},m_{j}\right)+\left(1-\mu \right)\,F\,M\,\left(m_{i},m_{j}\right),o\,t\,h\,e\,r\,w\,i\,s\,e \end{array}\right.$$

(5) $$D\,S\,\left(d_{i},d_{j}\right)=\left\{ \begin{array}{c} G\,D\,\left(d_{i},d_{j}\right),i\,f\,F\,D\,\left(d_{i},d_{j}\right)=0\\ \mu \,G\,D\,\left(d_{i},d_{j}\right)+\left(1-\mu \right)\,F\,D\,\left(d_{i},d_{j}\right)\;o\,t\,h\,e\,r\,w\,i\,s\,e \end{array}\right.$$

where μ is a control parameter for Gaussian similarity and functional similarity ranging from 0 to 1.

### 3.3. Graph convolutional network

In recent years, graph convolutional network as effective graph neural network model is widely applied in various fields with different tasks, such as node/graph classification, graph clustering and link prediction. The underlying idea of GCN is to learn node low-dimensional representations by aggregating node information from neighbors in a convolutional fashion while preserving graph structural information (Kipf and Welling, 2017; Zhang S. et al., 2019; Yue et al., 2020). Specifically, given a heterogeneous graph, the message propagation rule of GCN is expressed as:


(6) $$H^{\left(l+1\right)}=f\,\left(H^{\left(l\right)},G_{H\,N}\right)=t\,a\,n\,h\,\left(D^{-\frac{1}{2}}\,G_{H\,N}\,D^{-\frac{1}{2}}\,H^{\left(l\right)}\,W_{G\,C\,N}^{\left(l\right)}\right)$$

where *H*^(*l*)^ represents the node embedding at the *l*th layer, $W_{G\,C\,N}^{\left(l\right)}$ is the trainable weight matrix for the *l*th graph convolutional layer. *tanh* is a nonlinear activation function. *D* is the degree matrix of *G*_*HN*_. *G*_*HN*_ ∈ ℝ^(*nd* + *nm*)×(*nd* + *nm*)^ is consisted of adjacent matrix *A* and two similarity matrices (GH⁢N=[β⁢D⁢S*AATβ⁢M⁢S*]). D⁢S* and M⁢S*are normalizations of *DS* and *MS*, β is a penalty factor used to control the contribution value of the similarity matrix in *G*_*HN*_. The initialized embedding of the graph is denoted as $H^{\left(0\right)}\;=\;\left[\begin{array}{cc} 0 & A\\ A^{T} & 0 \end{array}\right]$.

### 3.4. Graph attention network

The graph attention network is another hot network architecture with the assumption that the node representation contributed from node neighbors is diverse (Veličković et al., 2018; Yu et al., 2021). After performing graph convolutional operation, the node representations can be learned from the network structure. Thereafter, we introduce the graph attention layers to improve the node representations based on GAT, focusing on the contributions of import node neighbors for node representation learning. Specifically, there are two steps: achieving the attention distribution and averaging representations with the corresponding distribution. More definitions are described as follows:


(7) $$e_{i\,j}^{\left(l\right)}=relu\left({\vec{a}}^{T}\left[W_{G\,A\,T}^{\left(l\right)}h_{i}^{\left(l\right)}||W_{G\,A\,T}^{\left(l\right)}h_{j}^{\left(l\right)}\right]\right)$$

(8) $$Z_{i}^{\left(l\right)}=\sum_{j\in N_{i}}a\,t\,t_{i\,j}^{\left(l\right)}\,h_{j}^{\left(l\right)}$$

where $e_{i\,j}^{\left(l\right)}$ indicates the importance of node *j* to node *i* in the *l*th layer, $h_{i}^{\left(l\right)}$ is the node representations derived from the *l*th graph convolutional layer. || is the concatenation operation, ${\vec{\text{a}}}^{T}$ is a weight vector, $W_{G\,A\,T}^{\left(l\right)}$ is a shared weight matrix, *relu* is a nonlinear activation function. $Z_{i}^{\left(l\right)}$ represents the representation of node *i* by averaging representations of its neighbor nodes with normalized attention distribution. $e_{i\,j}^{\left(l\right)}$ is normalized as $a\,t\,t_{i\,j}^{\left(l\right)}=\frac{e\,x\,p\,\left(e_{i\,j}^{\left(l\right)}\right)}{\sum_{c\in N_{i}}e\,x\,p\,\left(e_{i\,c}^{\left(l\right)}\right)}$, *N_i_* is the neighborhood of node *i* in the graph.

### 3.5. Decoder for microbe-disease association

We attain the learned feature representations *Z_m_* for microbes and **Z**_*d*_ for diseases from the output of GAT. Inspired by the work of Du et al. (2022), we reconstruct an association score matrix for microbe-disease associations (Equation 9) and define the local loss function which can dynamically reduce the weight of easily distinguished samples and make the distribution of loss function balanced (Lin et al., 2020) (Equation 10).


(9) $$\hat{A}=s\,i\,g\,m\,o\,i\,d\,\left(Z_{d}\,W^{’}\,Z_{m}^{T}\right)$$

(10) $$\ell_{f\,l}=\sum_{\left(i,j\right)\in \Omega^{+}\,⋃\Omega^{-}}\psi \,\left({\hat{A}}_{i\,j},A_{i\,j}\right)$$

where *W*′ is a trainable matrix, *sigmoid* is a nonlinear activation function. Ω^+^ and Ω^−^ denote the positive and negative sample sets, respectively. Moreover, we adopt the focal loss function ψ to solve the class imbalance. Focal loss (Lin et al., 2020) is based on binary cross-entropy and is a dynamically scaled cross-entropy loss.


(11) $$\psi =\left\{ \begin{array}{c} -\alpha \,{\left(1-{\hat{A}}_{i\,j}\right)}^{\gamma }\,l\,o\,g\,\left({\hat{A}}_{i\,j}\right),i\,f\,A_{i\,j}=1\\ -\alpha \,{\left({\hat{A}}_{i\,j}\right)}^{\gamma }\,l\,o\,g\,\left(1-{\hat{A}}_{i\,j}\right),o\,t\,h\,e\,r\,w\,i\,s\,e \end{array}\right.$$

where α is a weight parameter that controls the class imbalance between positive and negative samples, and γ is another weight parameter that controls the difficulty of sample classification. The Adam optimizer is used to minimize the loss (Kingma and Ba, 2015).

### 3.6. Parameter selection

There are several hyperparameters in MDAGCAN, such as the balance factor μ, the penalty factor β, the embedding dimension *k*, the initial learning rate *lr*, two weight parameters α and γ in focal loss, two dropout rates (node dropout *dp*_*n*_ and regular dropout *dp*_*r*_) and the iterations *epo*. These parameters consider different combinations from the ranges μ ∈ {0.1,0.3,0.5,0.7,0.9}, β ∈ {2,4,6,8,10}, *k* ∈ {32,64,128,256}, *lr* ∈ {0.05,0.005,0.0005,0.00005,0.000005}, α ∈ {0.1,0.2,0.3,0.4,0.5,0.6,0.7,0.8,0.9}, γ ∈ {1,2,3,4,5}, *dp*_*n*_ ∈ {0.1,0.3,0.5,0.7,0.9}, *dp*_*r*_ ∈ {0.1,0.3,0.5,0.7,0.9}, and *epo* ∈ {100,200,300,400,500,600}. After adjusting, we set the optimal parameters μ=0.5, β=8, *k* = 64, *lr* = 0.00005, α=0.1, γ=2, *dp*_*n*_ = 0.5, *dp*_*r*_ = 0.7, and *epo* = 500 for MDAGCAN in the following experiments.

## 4. Results

### 4.1. Performance evaluation

**FIGURE 2:**
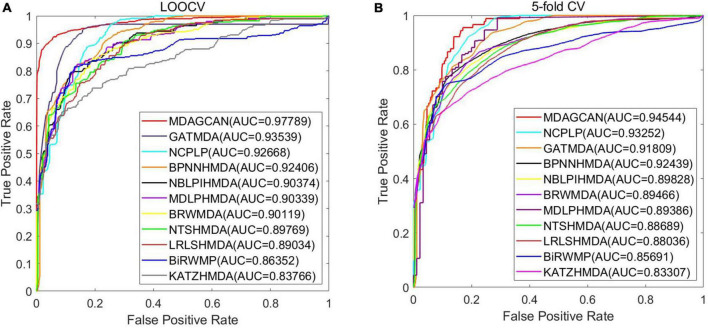
Prediction performance comparison between MDAGCAN and 10 baseline methods on the HMDAD dataset in LOOCV **(A)** and 5-fold CV **(B)**.

Until now, many methods have been proposed to predict microbe-disease association. However, there are no consistent results and poor performance attributed to the single dataset usage and improper model adoption. In this paper, we conduct different experiments on two datasets to fairly compare our method with the existing methods. First, under the evaluation framework of LOOCV and 5-fold CV, we compare our method (MDAGCAN) with 10 baseline methods on HMDAD dataset, such as the katz measure-based model KATZHMDA (Chen et al., 2017), the random walk models BiRWMP (Shen et al., 2018), NTSHMDA (Luo and Long, 2020) and BRWMDA (Yan et al., 2020), the conventional machine learning model LRLSHMDA (Wang et al., 2017), the matrix decomposition model MDLPHMDA (Qu et al., 2019), the network-based models NBLPIHMDA (Wang et al., 2019) and NCPLP (Yin et al., 2020), the neural network model BPNNHMDA (Li et al., 2020) and GATMDA (Long et al., 2021). Under the evaluation framework of LOOCV and 5-fold CV, MDAGCAN obtains the highest AUC values of 0.9778 and 0.9454, and has 4.25, 2.73% higher than the graph attention network method GATMDA, and 5.12, 1.29% better than the network consistency projection method NCPLP, respectively. All results are shown in Figure 2.

Besides, we perform the disease horizontal test, in which four-fifths of disease rows of the association matrix are randomly selected as the train set and the rest as the test set. Similarly, the microbe vertical test is also carried out in the columns of the association matrix. In the end, our method obtains AUC values of 0.8674 ± 0.0175 and 0.9290 ± 0.0143 on two tests, respectively. At the same time, we also compare MDAGCAN to other methods with different assessment metrics, such as F1 Score, Accuracy, Sensitivity and Specificity. More results are shown in Tables 2, 3. Obviously, the predictive effect of the microbe vertical test is better than the disease horizontal test due to the large degree difference of the disease node. When a disease with a large degree is used as the test set, the training set will contain less information, which will affect the prediction performance. The horizontal/vertical test suggests that our method achieves excellent performance, and is more suitable to predict new diseases and microbes.

**TABLE 2:** Performance comparison between 10 baseline methods and MDAGCAN under horizontal test for diseases in 5-fold CV on HMDAD dataset.

Methods	AUC	F1 Score	Accuracy	Sensitivity	Specificity
KATZHMDA	0.2625 ± 0.0777	0.5234 ± 0.1151	0.1649 ± 0.0371	0.3630 ± 0.1117	0.1636 ± 0.0377
BiRWMP	0.7345 ± 0.0418	**0.8161 ± 0.0789**	0.7637 ± 0.1030	0.6966 ± 0.1100	0.6936 ± 0.1049
LRLSHMDA	0.3794 ± 0.1462	0.5629 ± 0.1338	0.4029 ± 0.3159	0.4032 ± 0.1266	0.4022 ± 0.3171
NTSHMDA	0.4396 ± 0.1082	0.5032 ± 0.1151	0.4147 ± 0.2086	0.3434 ± 0.0966	0.4152 ± 0.2090
BRWMDA	0.3829 ± 0.0825	0.5769 ± 0.3827	0.3318 ± 0.1231	0.5114 ± 0.4092	0.3292 ± 0.1256
MDLPHMDA	0.4498 ± 0.1240	0.6403 ± 0.1234	0.3734 ± 0.3990	0.4833 ± 0.1399	0.3713 ± 0.4017
NBLPIHMDA	0.3846 ± 0.1316	0.5978 ± 0.1496	0.2481 ± 0.1841	0.4430 ± 0.1602	0.2468 ± 0.1849
BPNNHMDA	0.6166 ± 0.1743	0.7129 ± 0.1619	0.4321 ± 0.1506	0.6732 ± 0.2292	0.4289 ± 0.1522
NCPLP	0.8230 ± 0.0372	0.7883 ± 0.0088	0.7261 ± 0.0552	**0.8771 ± 0.0173**	0.7252 ± 0.0548
GATMDA	0.4586 ± 0.0195	0.4647 ± 0.0548	0.7591 ± 0.0509	0.5050 ± 0.0520	0.7573 ± 0.0523
MDAGCAN	**0.8674 ± 0.0175**	0.7367 ± 0.0865	**0.7826 ± 0.0545**	0.8539 ± 0.0261	**0.7810 ± 0.0540**

The best results are marked in bold and the second-best results are underlined.

**TABLE 3:** Performance comparison between 10 baseline methods and MDAGCAN under vertical test for microbes in 5-fold CV on HMDAD dataset.

Methods	AUC	F1 Score	Accuracy	Sensitivity	Specificity
KATZHMDA	0.8756 ± 0.0484	0.8456 ± 0.0263	0.8641 ± 0.0418	0.7828 ± 0.0423	0.8645 ± 0.0420
BiRWMP	0.8993 ± 0.0071	0.8549 ± 0.0579	0.8177 ± 0.1040	0.8190 ± 0.0987	0.8159 ± 0.1057
LRLSHMDA	0.8465 ± 0.0258	0.8267 ± 0.0499	0.8964 ± 0.0701	0.7064 ± 0.0561	0.8979 ± 0.0710
NTSHMDA	0.8465 ± 0.0258	0.8430 ± 0.0499	0.8857 ± 0.0742	0.7318 ± 0.0758	0.8869 ± 0.0754
BRWMDA	0.8657 ± 0.0309	0.7985 ± 0.0493	0.9061 ± 0.0049	0.6673 ± 0.0670	**0.9438 ± 0.0053**
MDLPHMDA	0.8019 ± 0.0288	0.8061 ± 0.0238	0.8470 ± 0.0473	0.6759 ± 0.0332	0.8484 ± 0.0478
NBLPIHMDA	0.8384 ± 0.0417	0.7968 ± 0.0496	**0.9280 ± 0.0034**	0.6651 ± 0.0705	0.9302 ± 0.0039
BPNNHMDA	0.9057 ± 0.0112	0.8653 ± 0.0485	0.8739 ± 0.0452	0.8307 ± 0.0830	0.8744 ± 0.0462
NCPLP	0.9184 ± 0.0093	0.9058 ± 0.0174	0.8204 ± 0.0440	0.8533 ± 0.0327	0.8194 ± 0.0445
GATMDA	0.9063 ± 0.0111	0.6917 ± 0.0263	0.8644 ± 0.0235	0.9091 ± 0.0214	0.8636 ± 0.0238
MDAGCAN	**0.9290 ± 0.0143**	**0.9062 ± 0.0401**	0.8559 ± 0.0410	**0.9232 ± 0.0159**	0.8549 ± 0.0418

The best results are marked in bold and the second-best results are underlined.

In order to validate the robustness of methods, we perform contrast experiments on dataset MASI. The experimental results show that our method also reaches the best average AUC (0.8730 ± 0.0036), accuracy (0.7996 ± 0.0157) and specificity (0.7691 ± 0.0142) compared with the state-of-the-art methods (Table 4).

**TABLE 4:** Performance comparison between 10 baseline methods and MDAGCAN in 5-fold CV on MASI dataset.

Methods	AUC	F1 Score	Accuracy	Sensitivity	Specificity
KATZHMDA	0.6869 ± 0.0160	0.7371 ± 0.0382	0.6048 ± 0.0556	0.7133 ± 0.0685	0.6026 ± 0.0580
BiRWMP	0.7370 ± 0.0228	0.7285 ± 0.0366	0.7616 ± 0.0260	0.7062 ± 0.0466	0.7627 ± 0.0272
LRLSHMDA	0.7724 ± 0.0115	0.8169 ± 0.0272	0.6453 ± 0.0366	0.8378 ± 0.0487	0.6413 ± 0.0383
NTSHMDA	0.7861 ± 0.0152	0.8490 ± 0.0273	0.7262 ± 0.0538	0.7523 ± 0.0520	0.7257 ± 0.0557
BRWMDA	0.8128 ± 0.0174	0.8681 ± 0.0422	0.7488 ± 0.0506	0.7658 ± 0.0378	0.7485 ± 0.0523
MDLPHMDA	0.8324 ± 0.0156	0.8755 ± 0.0293	0.7638 ± 0.0404	0.8099 ± 0.0547	0.7629 ± 0.0421
NBLPIHMDA	0.8209 ± 0.0140	**0.8818 ± 0.0187**	0.7311 ± 0.0569	0.7997 ± 0.0520	0.7297 ± 0.0590
BPNNHMDA	0.8049 ± 0.0133	0.8065 ± 0.0424	0.6774 ± 0.0552	0.8246 ± 0.0596	0.6744 ± 0.0574
NCPLP	0.7824 ± 0.0131	0.8128 ± 0.0217	0.6596 ± 0.0286	0.8528 ± 0.0461	0.6556 ± 0.0300
GATMDA	0.8206 ± 0.0173	0.7534 ± 0.0243	0.7642 ± 0.0448	**0.8794 ± 0.0400**	0.7619 ± 0.0463
MDAGCAN	**0.8730 ± 0.0036**	0.7840 ± 0.0135	**0.7996 ± 0.0157**	0.8411 ± 0.0206	**0.7691 ± 0.0142**

The best results are marked in bold and the second-best results are underlined.

### 4.2. Predicting associated microbes for liver cirrhosis and epilepsy

Furthermore, we validate the prediction performance of MDAGCAN on two datasets HMDAD and MASI for two common diseases, i.e., liver cirrhosis and epilepsy. In this study, to identify the potential microbe-disease pairs, we remove all known microbe-disease associations, and select the top 20 microbes based on the ranking scores as the highly associated entities with the queried disease. Results show that 16 and 17 out of the top 20 predicted microbes for liver cirrhosis and epilepsy are verified by published literatures, respectively. Top-20 predicted candidate liver cirrhosis-related and epilepsy-related microbes also are listed in Tables 5, 6.

**TABLE 5:** Prediction results of top-20 liver cirrhosis-related microbes.

Rank	Microbe	Evidence	Rank	Microbe	Evidence
1	*Clostridium difficile*	PMID: 26440041	11	*Clostridium leptum*	PMID: 24564202
2	*Helicobacter pylori*	PMID: 9365129	12	*Clostridiales*	PMID: 31726747
3	*Staphylococcus aureus*	PMID: 30253652	13	*Bifidobacterium*	PMID: 29806520
4	*Clostridium coccoides*	Unconfirmed	14	*Escherichia coli*	PMID: 36207946
5	*Staphylococcus*	PMID: 25518533	15	*Bacteroides vulgatus*	PMID: 23333527
6	*Actinobacteria*	PMID: 32265857	16	*Enterococcus*	PMID: 36035413
7	*Clostridia*	PMID: 30661942	17	*Bacteroides ovatus*	Unconfirmed
8	*Stenotrophomonas maltophilia*	PMID: 35755768	18	*Bacteroides uniformis*	PMID: 33348106
9	*Burkholderia*	Unconfirmed	19	*Prevotella*	PMID: 32414035
10	*Betaproteobacteria*	Unconfirmed	20	*Klebsiella*	PMID: 36147601

**TABLE 6:** Prediction results of top-20 epilepsy-related microbes.

Rank	Microbe	Evidence	Rank	Microbe	Evidence
1	Prevotellaceae	PMID: 35250450	11	Faecalibacterium	PMID: 35069460
2	Firmicutes	PMID: 35250450	12	Coprococcus	PMID: 6699268
3	Clostridiales	PMID: 30007242	13	Erysipelotrichaceae	PMID: 33415132
4	Enterobacteriaceae	PMID: 35069460	14	Clostridium	PMID: 6699268
5	Ruminococcaceae	PMID: 30007242	15	Rikenellaceae	PMID: 30007242
6	Clostridia	Unconfirmed	16	Bacteroidetes	PMID: 30007242
7	Bacteroidaceae	Unconfirmed	17	Ruminococcus	PMID: 6699268
8	Porphyromonadaceae	Unconfirmed	18	Streptococcus	PMID: 35250450
9	Roseburia	PMID: 31646147	19	Actinobacteria	PMID: 35250450
10	Lachnospiraceae	PMID: 30007242	20	Klebsiella	PMID: 34234109

Liver cirrhosis is a common degenerative disease of the liver, caused by one or more factors such as genetics, viruses and drugs, and has a high mortality rate. In our prediction result, *Clostridium difficile* is the most associated with liver cirrhosis which is the top of the ranking list. *Clostridium difficile* infection is one of the factors leading to liver cirrhosis and is widely used to perform fecal microbial transplantation for treating liver cirrhosis (Olmedo et al., 2019). Meanwhile, Clostridiales ranked twelfth is generally considered to be beneficial bacteria, while Staphylococcus ranked fifth is the genus of pathogenic bacteria Staphylococcaceae (Bhat et al., 2016). Except for the microbes confirmed by literatures, we find four microbes, including *Clostridium coccoides*, Burkholderia, Betaproteobacteria, Bacteroides ovatus, which are not directly reported the association with liver cirrhosis. There is a report that *Clostridium coccoides* appears increased abundance in patients with nonalcoholic steatohepatitis (NASH), which leads to liver fibrosis and develops into liver cirrhosis. In other words, they may be the new biomarkers for liver cirrhosis (Mouzaki et al., 2013).

Epilepsy is another common chronic neurological disorder around the world. Recent researches demonstrate that epilepsy patients tend to have dysbiosis or imbalance of gut microbial composition (Dong et al., 2022). Prevotellaceae, Actinobacteria and Streptococcus appear higher abundance compared to the healthy control group, and Firmicutes appears in the inverse pattern, where they are all ranked in our predicted top 20 score list. In addition, Clostridia, ranked sixth in the score list, is less reported about epilepsy, but Clostridium spp appears increased relative abundance in autism spectrum disorder (ASD) (Borghi and Vignoli, 2019), where ASD and epilepsy maybe have the same heredity and physiopathologic mechanism (Mei et al., 2017). The two rarely reported microbes for epilepsy are Bacteroidaceae and Porphyromonadaceae. But there is evidence that Bacteroidaceae is depleted after traumatic brain injury (Rogers et al., 2022) and the decrease of Porphyromonadaceae is closely linked to schizophrenia (Juckel et al., 2021). In the future, their important role in epilepsy will be further verified by wet experiments. In conclusion, results demonstrate that our method can effectively predict potential microbes for given diseases, which facilitates disease diagnosis and prevention.

## 5. Discussion

Over the last decade, increasing researchers pay more attention to the gut-liver-brain axis. The gut-liver-brain axis refers to the bidirectional relationship between the gut and its microbiota, the liver, and the brain, resulting from integrating signals generated by dietary, genetic, and environmental factors (Rocco et al., 2021). Growing evidences have emerged to consider the microbiota-gut-liver-brain axis as a comprehensive approach for better understanding diseases pathophysiology (Fuenzalida et al., 2021).

Figuring out the interactions between microbes and diseases provides a new way to diagnose and treat diseases. However, experimental identification of microbe-disease associations is time-consuming, laborious and expensive. The development of high-throughput sequencing technology has made it possible to explore the association between microbes and diseases on a large scale. In this paper, we present a deep learning framework based on the graph convolutional attention network. We integrate microbe similarity network, disease similarity network and known microbe-disease associations into a heterogeneous network. Then, we encode and learn the node feature information from its neighbors and itself via multiple graph convolutional layers and graph attention layers. Finally, MDAGCAN reconstructs the unobserved microbe-disease associations through a bilinear decoder. Comprehensive experiments demonstrate that our method MDAGCAN is promising and reliable to identify disease-related potential target microbes.

In addition, we further apply the microbe-disease association prediction model to predict liver cirrhosis and epilepsy-associated microbes and to find out the top 20 microbial candidates associated with them. Meanwhile, the indirect validation indicates that the remaining microbes are also associated with liver cirrhosis and epilepsy, respectively. They may be novel prospective biomarkers that require further experimental validation. Accumulating studies have revealed that epilepsy is associated with increased mortality in liver cirrhosis, but the underlying mechanism is still not known. Our analysis results display that there are four common microbes in the top 20 ranking score lists from liver cirrhosis and epilepsy, i.e., Actinobacteria, Clostridia, Clostridiales and Klebsiella. It is reported that the relative abundances of Actinobacteria and Klebsiella both increase in patients with liver cirrhosis and epilepsy compared with healthy controls (Chen et al., 2020; Lin et al., 2021; Dong et al., 2022; Zhou et al., 2022). Clostridiales with decreased abundance is strongly associated with the severity of liver cirrhosis and the seizure of epilepsy (Zhang et al., 2018; Fukui, 2019). Also, Clostridia appears inverse abundance pattern in liver cirrhosis and epilepsy patients (Zhang L. et al., 2019). Moreover, Actinobacteria produces SCFAs through metabolic pathways. SCFAs are vital components in the microbiota-gut-brain axis affecting the immune and endocrine systems through involvement in gut-brain signal pathways (Gong et al., 2021; Phillips-Farfan et al., 2021). Klebsiella and Clostridiales produce an extracellular toxic complex via metabolic pathways whose main component is lipopolysaccharide (LPS). LPS release mainly affects the inflammatory response in the whole organism and the gut-liver-brain communication (Ahluwalia et al., 2016; Boeri et al., 2019). In conclusion, the gut microbe is possible as a bridge to understand the pathogenesis of liver cirrhosis and epilepsy.

Although several experiments show that our method performs well in predicting new associations, there are still some limitations. On the one hand, the known microbe-disease associations are insufficient to attain better prediction performance due to data imbalance and sparsity. On the other hand, MDAGCAN lacks a wealth of prior biological knowledge like microbial phylogeny, microbial gene sequencing and disease semantic information to improve predictive performance. In the future, we will make further research and efforts to address these shortcomings.

## Data availability statement

The original contributions presented in this study are included in the article/supplementary material, further inquiries can be directed to the corresponding authors.

## Author contributions

LL implemented the experiments. ZLC analyzed the result. KS, LL, and SFF wrote the manuscript. KS and SFF designed the experiments and conducted the project. ZFW, HZC, and ZLC acquired the data and conceived the critical appraisal of the method. All authors read and approved the final manuscript.

## Footnotes

1
http://www.cuilab.cn/hmdad

2
http://www.aiddlab.com/MASI/

3
https://string-db.org
